# Macroscopic Viscosity of Polymer Solutions from the Nanoscale Analysis

**DOI:** 10.1021/acsapm.1c00348

**Published:** 2021-04-12

**Authors:** Airit Agasty, Agnieszka Wisniewska, Tomasz Kalwarczyk, Kaloian Koynov, Robert Holyst

**Affiliations:** † Department of Soft Matter, Institute of Physical Chemistry, Polish Academy of Science, Kasprzaka 44/52, 01-224 Warsaw, Poland; ‡ Max Planck Institute for Polymer Research, Ackermannweg 10, 55128 Mainz, Germany

**Keywords:** macroscopic viscosity, effective viscosity, length scale, rheometry, polydispersity, activation energy

## Abstract

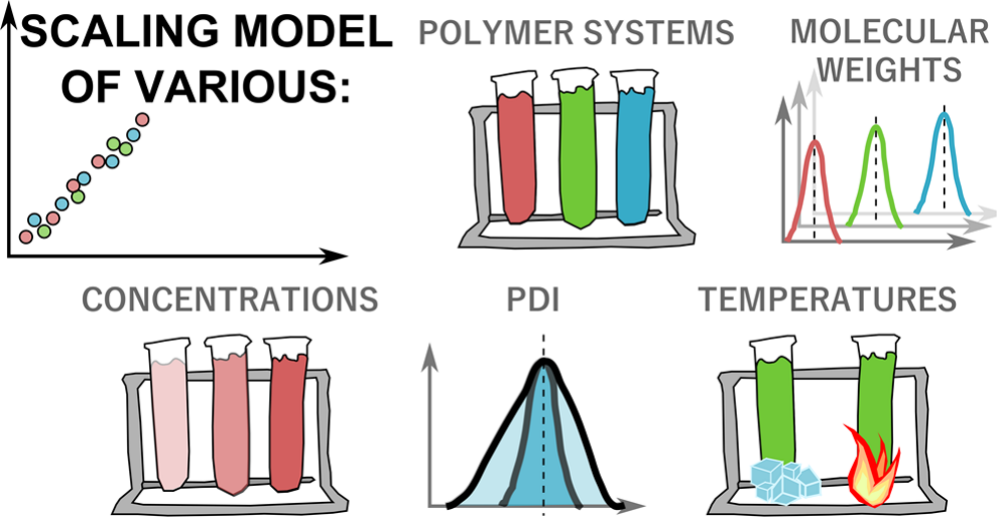

The effective viscosity in polymer solutions probed by diffusion of nanoparticles depends on their size. It is a well-defined function of the probe size, the radius of gyration, mesh size (correlation length), activation energy, and its parameters. As the nanoparticle’s size exceeds the radius of gyration of polymer coils, the effective viscosity approaches its macroscopic limiting value. Here, we apply the equation for effective viscosity in the macroscopic limit to the following polymer solutions: hydroxypropyl cellulose (HPC) in water, polymethylmethacrylate (PMMA) in toluene, and polyacrylonitrile (PAN) in dimethyl sulfoxide (DMSO). We compare them with previous data for PEG/PEO in water and PDMS in ethyl acetate. We determine polymer parameters from the measurements of the macroscopic viscosity in a wide range of average polymer molecular weights (24–300 kg/mol), temperatures (283–303 K), and concentrations (0.005–1.000 g/cm^3^). In addition, the polydispersity of polymers is taken into account in the appropriate molecular weight averaging functions. We provide the model applicable for the study of nanoscale probe diffusion in polymer solutions and macroscopic characterization of different polymer materials via rheological measurements.

## Introduction

Complex liquids such as polymer solutions contain internal length scales (e.g., the radius of gyration or correlation length) that influence their rheological properties. Because of this internal structure, the viscosity of polymer solution depends on the flow length scale. At length scales below the polymer coil’s size, the viscosity is close to that of the solvent. Far above the radius of gyration, the macroscopic viscosity governs the flow. In previous works, we studied the diffusion of nanoprobes in hexaethylene-glycol-monododecyl-ether and PEG/PEO solutions in water for a wide range of nanoprobes sizes (0.28–190.00 nm).^1,2^ The size of the probe sets the length scale at which we probe the viscosity. We determined the effective viscosity experimentally as a function of probe size, *r*_p_, from the diffusion coefficient of nanoprobes, *D*
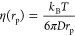
1while the following theoretical equation gave the effective viscosity
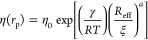
2Here, η(*r*_p_) is the effective viscosity experienced by the nanoprobes (in units of Pa·s), η_0_ is the solvent viscosity (also in units of Pa·s), *R* is the gas constant, *T* is the temperature in the absolute scale, *k*_B_ is the Boltzmann constant, *a* is a structural parameter of the order of unity, γ is the effective activation energy of the solution (in kJ/mol), and ξ is the correlation length (in nm). *R*_eff_ is the length scale given by^3−5^
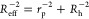
3where *R*_h_ is the hydrodynamic radius of polymers in solution. For large probes, *R*_h_ ≪ *r*_p_, eq 2 reduces to the macroscopic viscosity η_macro_ of the polymer solution
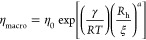
4where η_macro_ is the viscosity experienced at the macroscale. We have successfully applied this approach to various complex liquids: colloidal solutions, protein solutions, micellar solutions, cytoplasm of HeLa cells, and *Escherichia coli*.^2,5−7^ In this paper, we apply the model to three different polymer solutions in a wide range of molecular masses (24–300 kg/mol), temperatures (283–303 K), and concentrations (0.005–1.000 g/cm^3^).

The literature provides many different macroscopic viscosity models of polymer solutions. The earliest example of such a scaling model was that proposed by Huggins,^8^ and it was based on the specific viscosity of very dilute polymer solutions. When simplified, the Huggins relationship was of the form
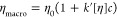
5with *k*′ being an empirically determined polymer-solvent constant and *c* being the concentration of polymer solutions. The intrinsic viscosity, [η], is a measure of the concentration dependence of the viscosity. Such a theory was based on determining the viscosity at very low dilutions and also corroborated by others such as Martin; Schulz and Blaschke; Fikentscher and Mark; de Jong, Kruyt, and Lens; and Baker.^9−14^ All of these different viscosity models are essentially of the same form as the one above by Huggins, with different empirical constants of the same order of magnitude. Crucially, these works focused on the dependence of the viscosity of polymer solutions on their concentrations. Further works by Barry,^15^ Korolev et al.,^16^ and Warrick et al.^17^ developed empirical viscosity scaling models relating the dependence of viscosity on the molecular weight of the polymers. The observed relationships were based on the well-defined Mark–Houwink equation^18,19^ developed around the same period of time. It correlated the viscosity to the molecular weight as
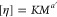
6where *K* is another empirical constant, similar to the empirical parameter developed by Huggins, and *a*′ is the Mark–Houwink parameter. The difference was the use of intrinsic viscosity as opposed to the specific viscosity in their descriptions.

Further work on scaling concepts was carried out by de Gennes that utilized the models by Huggins and Flory.^20^ In these developments, the concepts of entanglements in polymer solutions and their mathematical definitions were obtained. The parameter ξ from eq 4, defined as the correlation length, was developed in these models to portray the changes in the gradual entangling of polymer chains in solution. Polymer-solvent characteristics as well as the dimensional characteristics of the polymer chains with regard to their orientation in the solution were also investigated. Application of the generalized Zimm models^21,22^ led to an establishment of the power law equations relating the polymer coil dimensions to the molecular weight of the polymers as
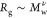
7The value of *ν* is determined from the mean-field theory and is indicative of the repulsive excluded-volume interactions. As shown by Flory^23^ in the mean-field model, ν = 0.6 for polymers in good solvents. The de Gennes scaling and similar related models^24,25^ also managed to identify changes to the dimensions due to increase in the concentration of the solutions, specifically due to excluded-volume effects at higher concentrations. The idea of different concentration zones such as dilute, semidilute, and concentrated were observed, but most experimental results driving the theory were limited to semidilute polymer solutions. Polymer scaling theories developed later that included all three concentration zones, such as the “fuzzy-cylinder” approach of Sato et al.^26,27^ managed to provide good relationships between the zero-shear polymer viscosity and its variations of concentration and molecular weights. But this model was based on the previously described eq 1 for diffusion characteristics. As mentioned before, the SSE equation can fail by orders of magnitudes in many cases when applied to measurement of nanoviscosities.

Over the years, many such scaling theories have been applied with their underlying theory driven by some of the above-mentioned models. Some are restricted by concentration limits (usually applied in dilute or semidilute zones), while others are limited in their applicability when effects of dimensional changes versus concentration, temperature, activation energies for polymer-solvent systems, or distribution range of molecular weights are considered. Type of polymer and solvent also plays a part in such models, and often the practical application of such theory is limited. More importantly, such models cannot traverse different length scales of the viscosity of complex systems, and therefore cannot be considered as universal.

The most important factor in the processing methods is the flow characteristics of the material, which is driven by the inherent structures and properties of both the polymer and the solvent.^5,28^ Such specificity of flow characteristics is crucial to polymer analysis, and it is determined by measuring their viscosities. Through our recent papers,^2,6,7,29,30^ we have analyzed the viscosity of polyethylene glycol/polyethylene oxide (PEG/PEO) solutions in water and polydimethylsiloxane (PDMS) solutions in ethyl acetate. Our analysis covered solutions across all characteristic concentration regimes: dilute, semidilute, and concentrated,^24,28,31^ as well as multiple temperature and molecular weight ranges.

Our previous discussions^29,30^ confirmed the importance of these parameters for different systems and the resultant viscosity-based characterization. However, one important underlying aspect integral to all of these parameters is the molecular weight of the polymers. Standard polymers with well-characterized narrow chain distributions are great from the perspective of theoretical developments. Practical applications, on the other hand, are limited to faster manufacturing processes and techniques, resulting in polymers with broader weight distributions. It is also crucial that the model is applicable on a larger scale, being suitable for a wide range of polymers and solvents as required.

In the present paper, we validate eq 4 for other common polymer systems through experimental data: hydroxypropyl cellulose (HPC) in water, polymethylmethacrylate (PMMA) in toluene, and polyacrylonitrile (PAN) in dimethyl sulfoxide (DMSO), and explain its applicability for commercial or standard polymers with diverse molecular weight distributions; clarify the reasons and means for the validity of this model regardless of the polydispersity of the polymer samples; and provide a final consolidated information about all of the different parameters in our models. Quantified scaling parameters, activation energy and its parameters, and coil dimensions are provided for all current and previous solution systems. Our method provides the possibility to use a length-scale-based polymer characterization technique, developed on viscosity measurements, and is effective for a wide variety of applications, both at the nanoscale and the macroscale.

## Materials and Methods

HPC of commercial molecular weights 80 and 100 kg/mol, and PAN of 150 kg/mol were obtained from Sigma-Aldrich. PMMA was obtained from in-house synthesis and in weights of 24 and 70 kg/mol. Solvents for each system were selected on the basis of Hildebrand solubility parameter values for good solvents^32,33^ and also bearing in mind the application range of each. Different concentration ranges for the solutions were prepared from 0.005 to 1.000 g/cm^3^. The solutions were stirred at 800 rpm for 1–2 days at room temperature for all of the polymers. Viscosity measurements at all temperatures and concentrations were performed using a Bohlin Gemini rheometer as well as a Malvern Kinexus Pro rheometer with a cone-plate and coaxial cylinder geometries. The dependence of viscosity on temperature was measured in the temperature range of 283–303 K. Temperature was controlled within ±0.1 K. Viscosity of dilute polymer solutions was close to the solvent viscosity. To provide more accurate data in this region, we performed experiments based on coaxial cylinder geometries. The viscosity of these solutions was measured at temperature intervals of 5 K. For the viscosity measurements at higher concentrations, the cone-plate geometry selected had an angular gradient of 0.02 radians. Shear rate was kept between 0.1 and 500 s^–1^ depending on the type of polymer analyzed, and the shear stress range was varied accordingly for the purpose of measurements. All measurements were performed at steady shear rates. Measurements for PAN in DMSO were carried out up to 288 K and not lower, since DMSO freezes below that point. The linear viscosity data obtained were extrapolated to get the zero-shear viscosity. This zero-shear viscosity was then used as the viscosity of the polymer solution (see Tables S1–S5 and Figure S1 in the Supporting Information). All viscosity data reported had no more than 10% of errors in the measurements.

The molecular weight as well as the polydispersity index (PDI) of the polymers were measured by gel permeation chromatography (GPC) measurements to obtain correct molecular weight distributions (Mw, Mn, Mz, avg, etc.) and the polydispersity of the samples (Table S6 and Figure S3 in the Supporting Information). The GPC measurements were performed with specific calibration for each system, depending upon the type of polymer and solvent. Measurements were performed with an Agilent Series 1260 device equipped with a PSS SECcurity pump and a PSS SECcurity RI refractive index detector. For the cellulose, 0.1 M NaCl–water solution was used as an eluent at a flow rate of 1.0 mL/min and at a temperature of 303 K. For PAN and PMMA, dimethylformamide (DMF) and toluene, respectively, were used as an eluent at a flow rate of 1.0 mL/min and at temperatures of 333 and 303 K, respectively. Dynamic light scattering (DLS) measurements were performed on a Malvern Zetasizer equipment to obtain the hydrodynamic radius of polymers of different molecular weights in dilute solutions (see Figure S4 in the Supporting Information).

## Results and Discussion

### Identification of Previously Defined Parameters for Each Polymer-Solvent Systems

Viscosity measurements were performed for every 5 K temperature increase in the range 283–303 K. As per our previously developed model,^29,30^ we obtain the crossover concentrations c* and c** for each polymer-solvent system using the equations
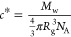
8and
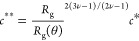
9where *R*_g_ denotes the gyration radius of the polymers, *N*_A_ is Avogadro’s number, *M*_w_ is the weight-average molecular mass of the polymer, and ν is a mean-field theory-based parameter, usually relating the gyration radius to the molecular weight. ν provides an indication of the repulsive excluded-volume interactions in the system. As per Flory,^23^ ν = 0.6 is applicable for polymers in good solvents ideally. However, experimental results do not always conform to the same, and quite a lot of studies go into identifying these values. For instance, Clasen et al.^34^ and thereafter Brumaud et al.^35^ have worked out differences of the relating coefficients and ν for obtaining the gyration radii for relatively monodisperse cellulose ether variants in water solutions. Based on such literature data, as well as taking into account the differences related to polymer synthesis and our own data fitting, the gyration radii of the polymers in our systems could be obtained as
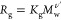
10and the details of the coefficients and exponents are listed in Table 1.

**Table 1:** Coefficients *K*_g_ and Exponents ν′ for Every New Solution System, Obtained against *M*_w_

solution system	*K*_g_	ν′
HPC-water	0.0272	0.542
PMMA-toluene	0.0275	0.546
PAN-DMSO	0.0255	0.530

**Figure 1:**
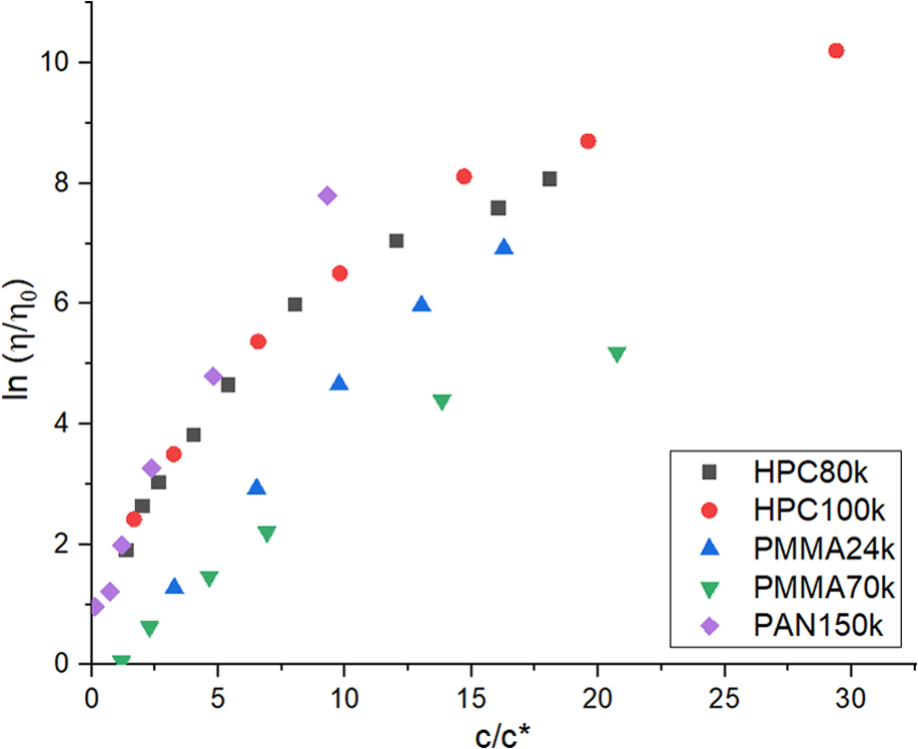
Results of relative viscosity measurements for polymer solutions plotted against ratio of concentration *c* to overlap concentration c*, at 298 K.

For all molecular weights and concentrations of the polymer systems, initially all of the viscosity data were plotted against the ratio of *c* to c*, according to the proposed general scaling theory of de Gennes. Here, both the parameters *c* and c* were represented in terms of the mass of the polymer per unit volume of the solvent. A clear dependence could be observed in Figure 1 as expected in theory.^2,36^ However, the data did not collapse on a single line, especially at very high concentrations. Thereafter, the viscosity scaling paradigm (eq 4) was applied, which was developed and perfected for different concentration systems before.^30^ Due to the fact that the powdered polymers had a saturation limit of dissolution in the solvents within our temperature range, calculations of c** showed us that the applied concentrations did not venture into the theoretical concentration ranges above c**, unlike before with PDMS-ethyl acetate. Therefore, all fitting was applied in the dilute and semidilute concentration ranges as done by Wiśniewska et al.^29^ on PEG/PEO-water solutions. HPC in water solutions starts to separate out beyond approximately 313 K and also shows slightly non-Newtonian behavior beyond 5% by weight concentrations in solution. HPC also has a maximum solubility of about 30% by weight in water, and thereafter shows completely non-Newtonian behavior as well. As such, experimental solution concentrations were always lower than the theoretically established concentrated zones, which was up to 27–30% by polymer weight. The same limits were also observed for PAN-DMSO and PMMA-toluene, and so all experimental concentrations were limited up to the semidilute concentration zones.

The exponent *a* in eq 4 is a parameter that changes discontinuously at the crossover to the different concentration regimes. For PEG/PEO-water solutions, *a* = β^–1^ for dilute regime and *a* = *R*_h_*R*_g_^–1^β^–1^ for semidilute regime of concentrations,^29^ where the exponent β is obtained from Flory’s mean-field theory.^23^ Parameter *a* is a characteristic for a specific polymer-solvent system, which provides information on the internal structure of any complex liquid.^36^ Crossovers between the three regimes of dilute, semidilute, and concentrated solutions lead to changes in the internal structures, and thereby changes in the value *a*.^28,37−39^ Fitting of eq 4 allows us to obtain the different values of *a* within acceptable deviations as provided in Table 2 for all polymer systems investigated so far.^5,30,40^

**Table 2:** Scaling Parameter *a* Values for Different Polymer Systems, in Different Concentration Regimes—Dilute (Dil), Semidilute (SDL), and Concentrated (Conc)

scaling parameter *a* values				
polymer system	Dil	SDL	conc	error
HPC-water	1.28	0.85		±0.02
PMMA-toluene	1.25	0.75		±0.02
PAN-DMSO	1.25	0.86		±0.02
PDMS-ethyl acetate	1.28	0.85	0.59	±0.02
PEG/PEO-water	1.29	0.78		±0.02

The values are in line with the available literature values for other polymer systems^34,35,41,42^ in dilute and semidilute systems, with the former reflected as such in good models for polymer systems developed by Wiśniewska et al.^5,29^ for PEG/PEO-water. As before with PDMS-ethyl acetate systems, the obtained data for *a* remain applicable for all of these polymer systems of all different molecular weights.

### Molecular Weight Averaging Function in Polydisperse Samples

**Figure 2:**
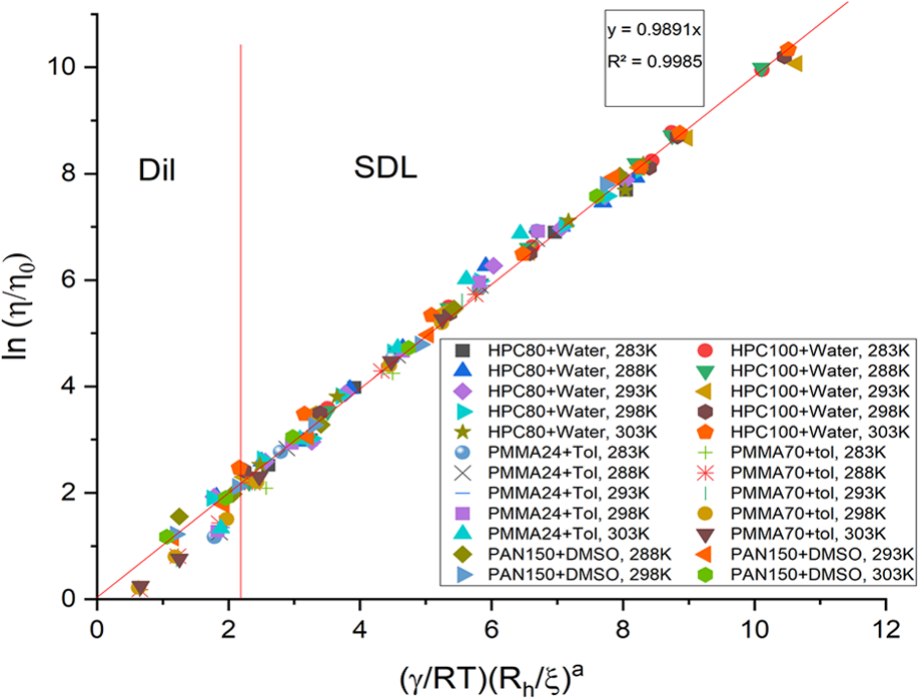
Viscosity scaling plots for all molecular weights of HPC-water, PAN-DMSO, and PMMA-toluene at all temperatures (283–303 K), plotted for calculations made with *M*_w_. The figure also shows the linearity obtained. More information is provided in Supporting Information Figure S5.

In our measurements, we obtain the weight-average molecular mass, *M*_w_, of the polymers through GPC and apply it to obtain the scaling model parameters. This method does not take into consideration the polydispersity of the polymers. Thus, the scaling obtained for our new polymer systems is not quite linear as shown in Figure 2 and needs a rectification of the averaging function employed for the polydispersed samples.

There are various methods utilized for obtaining the molecular weight of polymer chains. This is because the polymerization techniques lead to scattering of the molecular weights through different polymer chains due to the kinetics or thermodynamics of the reactions. The behavior of a polymer in solution or melt form is dependent on this distribution of the molecular weights (MWD). The need for obtaining a concept of mean or average of these MWDs is critical to relate the behavior of the polymer to its characteristic molecular weight. Theoretically, different methods are available in the literature for determining this average molecular weight,^43−46^ and they are employed through fractionation techniques such as GPC to obtain information about the different fractions. In reality, however, the separated fractions are only somewhat narrower in distribution than the rest of the polymers. These fractions are imagined as perfect and reported as such. Mathematically, the simplest method for obtaining the average molecular weight is simply an arithmetic mean of the molar masses of each macromolecule, and is known as the number-average molecular weight, *M*_n_. It is of the form
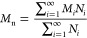
11The second averaging definition employed is known as the weight-average molecular weight, *M*_w_, and in this case, the sum of the product of the weight fraction to the molar mass of each species is considered. It is usually calculated by
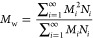
12*M*_n_ predicts the number of particles in each species present inside a system. *M*_w_ however provides information not only on the number of molecules of each species but also about their masses. The dispersity of polymer samples is obtained from the ratio of the above two mass indices, and is commonly known as the polydispersity index, PDI, of the sample. Therefore
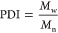
13The closer the value of this index to 1, the narrower the distribution of the molar mass fractions, and the more uniform the polymer chains. However, in common manufacturing processes, it is hardly ever possible to obtain such perfectly synthesized polymers. This leads to the PDI of polymers being greater than 1, and *M*_n_ < *M*_w_.

Another relative method of obtaining an average molecular weight from Chee^47^ involves measuring the intrinsic viscosities of dilute polymer solutions and using the Mark–Houwink–Sakurada (eq 6) relationship to obtain the viscosity average molar mass as
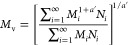
14where *a*′ is the Mark–Houwink (MH) parameter available for specific polymer-solvent systems. The Mark–Houwink (MH) equation can also be obtained when eq 4 is reduced to a generalized form. In fact, the scaling parameter *a* in eq 4 is of the same form as the MHS exponent *a*′ and replaces it in all of our calculations. *M*_v_ always takes up values in between *M*_n_ and *M*_w_.^45,48,49^ The distribution of molar masses can be depicted as in Figure S2 in the Supporting Information.

It is seen from Figure S2 that for all situations, *M*_n_ < *M*_v_ < *M*_w_. Using Taylor series expansion of eq 14, and disregarding the higher expansion terms of *a*, a simple linear equation relating *M*_w_ and *M*_v_ can be obtained as
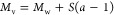
15where *S* is quantified as
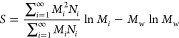
16On further investigation, it can be observed that the parameter *S* and *M*_w_ can be related through a digamma function of PDI, and it is of the form
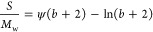
17Here, ψ(*x*) is the digamma function in (*x*), which in this scenario is the parameter *b*, and *b* itself is related to the PDI as
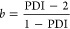
18It shows that in spite of most studies interchangeably using *M*_w_ and *M*_v_ for molecular weight information, it is not necessarily the case. In many situations, depending on polydispersity, the values can vary quite significantly. Crucially, it can be observed that eqs 17 and 18 cannot describe the case when PDI = 1, since *b* becomes undefined. Taking limits of eq 17, we find that PDI → 1, *S*/*M*_w_ → 0, and *M*_v_ → *M*_w_ in eq 15. The Schulz distribution function, on which eqs 17 and 18 are based, was derived from real systems, and in all such cases, *M*_n_ ⩽ *M*_w_. For the ideal perfect scenario where *M*_w_ = *M*_n_, there is no need for any model or relevant equations and the distribution function shown in Figure S2 collapses to a single straight line. As a result, in such a case, *M*_v_ is also the same as the other averaging functions of the molecular weight. Thus, the above equations are important as they allow for a means to obtain the different averaging indices of molecular weights based on its characteristics in real systems, which is more useful.

Polydispersity of commercial polymers is extremely relevant to their applicability. Previously, our models were applied for highly monodispersed standard polymers. In reality, it is rare for bulk production polymers to be monodisperse. As explained before through eqs 12–18, we obtain a means to make our base eq 4 useful for all kinds of polymer molecular weight distributions, no matter they are broad or narrow. As can be seen from our GPC experiments in the Supporting Information, the HPC polymers had high polydispersities of over 4, and even the polyacrylonitriles had a relatively higher polydispersity. Judging from eq 15, it can be seen that for monodisperse samples and PDI close to 1, the different molecular weight averages, *M*_w_ and *M*_v_, are identical and it does not matter which is used for quantitative measurements through the model. In such a case, it is more common to use the distribution which can be obtained more easily experimentally, such as through GPC. However, beyond a certain value of PDI, the gap between the distributions increases and using *M*_v_ for polydisperse sample is more reliable. It takes into account the input of different weight fractions of chains and provides an averaging function closer to the peak. As such, applying eqs 12–18, we can obtain *M*_v_ for the different polymer fractions with different molecular weights as shown in Table 3. It can be seen from Table 3 that in the case of PMMA systems, PDI is very low and can always be approximated as a monodispersed sample. However, there are still certain variations in the different molecular weight distributions, and its low effect is observed through the *S*/*M*_w_ ratios.

**Table 3:** *M*_v_ and *M*_w_ Relations for the Polymer Systems

polymer	*M*_w_ (g/mol)	PDI	S/*M*_w_	*M*_v_ (g/mol)
HPC80k	107 336	4.71	–0.442	95 475
HPC100k	146 690	4.82	–0.446	130 346
PMMA24k	25 063	1.08	–0.037	24 831
PMMA70k	73 416	1.14	–0.381	66 418
PAN150k	333 257	2.45	–0.324	306 266

For these calculations, the parameter *a* used is the same scaling parameter values for *a* in our dilute solution regions. It is in line with the theory as all of these measurements for *M*_w_ or intrinsic viscosity per the Mark–Houwink equations are performed at the dilute solution ranges. It maintains our analysis in line with the theory and shifts the averaging point to a better estimation. As can also be seen from Table 3, the difference in *M*_w_ and *M*_v_ for the highly monodisperse samples is extremely low (less than 5%) and therefore maintains the theory that they can be used interchangeably in all such calculations.

**Figure 3:**
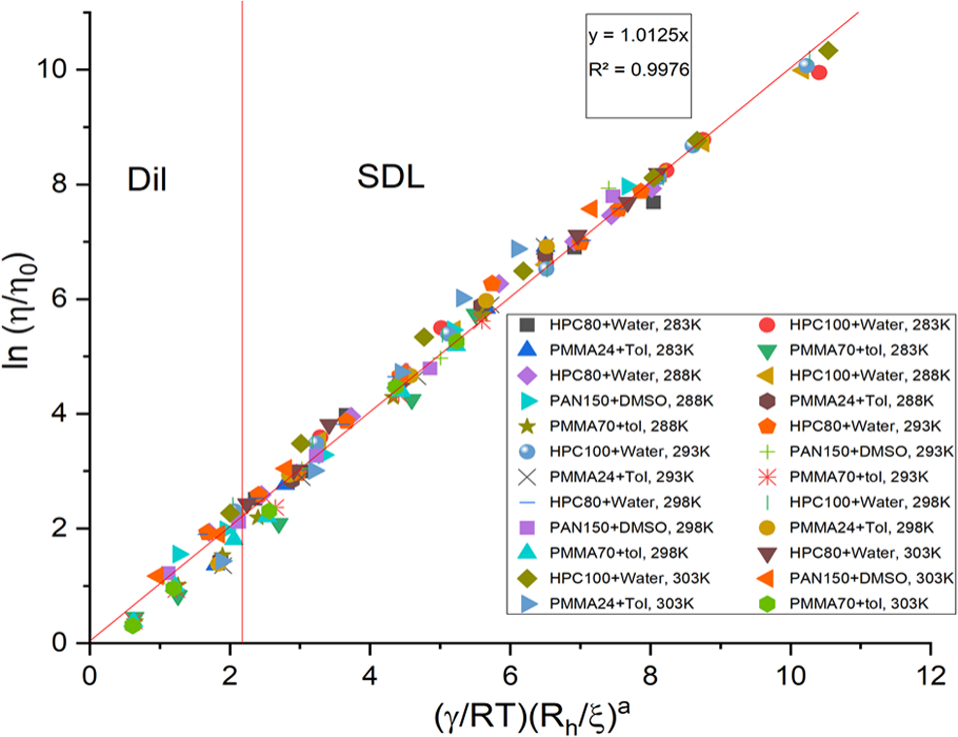
Viscosity scaling plots for all molecular weights of HPC-water, PAN-DMSO, and PMMA-toluene at all temperatures (283–303 K), plotted for calculations made with *M*_v_. The figure also shows the linearity obtained. More information is provided in Supporting Information Figure S6.

**Figure 4:**
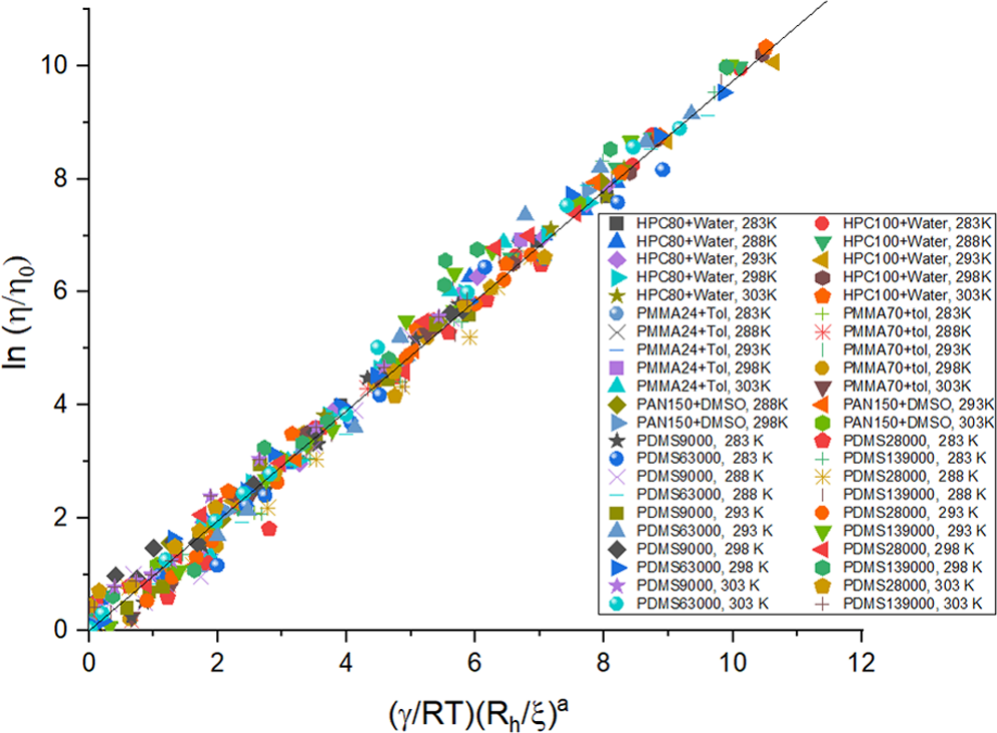
Viscosity scaling including the temperature dependence and the change of parameters at different crossovers. Measurements were performed on PDMS-ethyl acetate, HPC-water, PAN-DMSO, and PMMA-toluene, for different molecular weights (9–300 kg/mol), concentrations (0.001–8.000 g/cm^3^ for PDMS, 0.005–0.300 g/cm^3^ for HPC, PAN, and PMMA), and temperatures (283–303 K).

Reapplying *M*_v_ for *M*_w_ for HPC and PAN calculations provides us with an overall linear fitting as can be seen in Figure 3. All of the fitting curves are compared with the use of *M*_w_ (Figure 2) and *M*_v_ (Figure 3) separately, and the linearity of the curves is noted. It informs us that fitting with *M*_v_ is clearly a better choice than *M*_w_, and therefore should be considered in the case of polydisperse samples to obtain a more accurate picture. Figure 4 also provides a complete picture of the total linear scaling as observed from a compilation of the data from all of the polymer-solvent systems we have studied experimentally so far (including the data for PDMS-ethyl acetate).^30^

### Coil Dimensions *R*_h_ and *R*_g_ versus Concentration

As explained in our previous work,^5,30^ it is essential to identify the changes in the apparent polymer coil dimensions inside the solutions due to varying concentrations. One such parameter defining the mean distance between neighboring coils in entangled concentration zones is the correlation length ξ. It is defined as
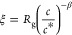
19As already mentioned, the exponent β can be derived from Flory’s mean-field theory.^23^ Generally, the gyration radius, *R*_g_, describes the isolated polymer coil blob size at all concentrations and is not the same as ξ.

Hydrodynamic radius of the coils, *R*_h_, on the other hand was initially measured at dilute concentrations through DLS. The data obtained, in conjunction with available literature empirical values, were fit through scaling eq 4 to obtain the relationship between the sizes and the molecular weights. Generally, both coil sizes (in units of nm) (*R*_h_ and *R*_g_) and molecular weights for long chains are related by the empirical power law equations of the form^50^ (see also eq 7)
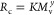
20where the parameter *R*_c_ is the general coil radius, which can be either the gyration or hydrodynamic radius, and the constants *K* and *y* have values specific to a polymer-solvent system.^51−53^ Such results focus on the effects of chain stiffness on the coil sizes at higher concentrations. The gyration radius, *R*_g_, is evaluated from eq 20, with some minor changes in the coefficient of the power law to compensate for the change in solvent and fitting from the experimental data. Power law relationships for the hydrodynamic radius, *R*_h_, for the three new polymer-solvent systems studied here were obtained from the DLS measurement data. Within the limits of empirical values available for other polymer systems^5,30,42^ as well as other polymer-solvent systems,^52−59^ the fit of *R*_h_ and *R*_g_ with eq 4 formulated the following relations for all our polymer systems as shown in Table 4. In these results, all radii in are nanometers and all molecular weights are in g/mol.

**Table 4:** Power Law Parameters of Hydrodynamic and Gyration Radii, *R*_h_ and *R*_g_, for Different Polymer Systems from Eq 20

**equation***R*_**h**_ = ***K*****’*****M***_**v**_*^**y**^***’**		
polymer system	*K*’	*y*’
HPC-water	0.0120	0.580
PMMA-toluene	0.0106	0.570
PAN-DMSO	0.0110	0.563
PDMS-ethyl acetate	0.0113	0.570
PEG/PEO-water	0.0145	0.571
**equation***R*_**g**_ = ***K″M***_**v**_*^**y**^***″**		
polymer system	*K***″**	*y***″**
HPC-water	0.0278	0.553
PMMA-toluene	0.0270	0.535
PAN-DMSO	0.0255	0.533
PDMS-ethyl acetate	0.0265	0.530
PEG/PEO-water	0.0215	0.583

At dilute concentrations, we assume a hard sphere model of the polymer coils, well separated from each other, and unaffected by any local fluctuations of the monomer density. Usually, it is as a result of focus on a specific region of polymer concentrations—in the dilute zone, or up to semidilute zone, and so on. In the dilute solutions, the polymer coils are separated and far away from each other.^24^ Interchain or intrachain interaction effects do not play any part with such low amount of coils in the solution, and so the coil dimensions remain unaffected by any slight change in concentrations. The ratio of the coil dimensions remain as such. Considering that previously established values of *R*_*h*_/*R*_*g*_ are approximately around 0.6 numerically for all polymers in good solvents,^60−62^ the relationship for all our polymer-solvent system maintains the same numerical state. When working with a whole range of polymer solutions from dilute up to polymer melt, it is vital to consider the relative changes that occur in the size of the polymer coils due to concentration changes. *R*_h_ and *R*_g_ provide us information regarding the hydrodynamic and static screening lengths as well.^24,63^ In the works of Daoud and Jannink,^24^ as also proposed by Cheng et al.,^50^ it is shown that the coil dimensions should decrease with concentration in the semidilute and concentrated ranges, due to screening effects of repulsive intrachain interactions as opposed to interchain interactions. Bennett et al.^63^ went further in a similar approach to extend the variation of hydrodynamic screening length fluctuations of polymers in higher-concentration solutions beyond c*. This approach predicts a decrease in the static and hydrodynamic screening lengths with increasing concentration. The ratio of the hydrodynamic to static screening lengths therefore increases with increasing concentration. Our viscosity data-based scaling agrees with the same principle of the concentration fluctuations in the semidilute zone. This leads to a slight increase in the coil dimensions in the semidilute zone and can be determined by the following proposed relationship
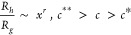
21with *x* being the mole fraction of the monomer in the solution. Our predicted exponents for *x* (approximately 0.053) for all our systems are shown in Table 5. The exponents for HPC are far lower than the other polymers,^59,64,65^ as HPC has a far stiffer chain and resists deformations. Consequently, it leads to far lower increase in sizes compared to the other polymers.

**Table 5:** Exponent *r* Defining the Dependence of Coil Size on Mole Fraction

polymer system	*r*
HPC-water	0.005
PMMA-toluene	0.043
PAN-DMSO	0.053
PDMS-ethyl acetate	0.053
PEG/PEO-water	0.041

The new polymer systems did not have concentrations that could reach the concentrated zones, and so, the size dependence is not determined unlike for PDMS-ethyl acetate.^30^ This is a general issue with most polymers in that their effective concentration zones usually lie in the semidilute concentration regions by theory. This also explains why most available scaling theories in the literature were usually developed for semidilute concentration regimes. The parameter R_h_ is crucial for obtaining the specific polymer-solvent relationship. Our fitted model provides the *R*_h_/*R*_g_ ratio, which indirectly also relates to hydrodynamic volume changes proportional to the viscosity as defined under the shear flow (obtained through eqs 4, 8, and 19). Crucially, instead of the various factors that influence the chain stiffness, we have tried to provide a simplified model that directly provides the size changes due to such stiffness effects.

### Activation Energy γ and Its Parameters

We developed an activation energy parameter γ,^5^ related through the rate theory of Eyring.^66,67^ It is based on overcoming the frictions occurring between the different molecular groups inside the solution for its flow to occur. We further expanded the study of this parameter to identify the exact nature of its components.^30^ Depending on the solution concentrations, the amount of frictional interactions inside the system varies, and so does the energy required for the flow of the viscous solution. Since there is always a certain amount of polymer present in our solutions, the total interaction parameter, γ, is assumed to be a sum of the weighted fractions of the different molecules inside the system. Thus, γ is defined as
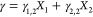
22where the subscripts 1 and 2 denote solvent and monomer, respectively, and *X* denotes the mole fraction of the corresponding component. By the same fitting applied to eq 4 and from the results depicted in Figure 4 before, we obtain estimates of the different activation energy parameters provided in Table 6.

**Table 6:** Activation Energy Parameters for All Polymer-Solvent Systems Studied in Eq 22

all γ are in units of kJ/mol		
polymer-solvent	γ_1,2_	γ_2,2_
HPC-water	4.20 ± 0.50	2.70 ± 0.30
PMMA-toluene	4.60 ± 0.70	3.10 ± 0.60
PAN-DMSO	4.30 ± 0.30	2.90 ± 0.25
PDMS-ethyl acetate	4.00 ± 0.50	2.75 ± 0.50
PEG/PEO-water	4.20 ± 0.50	2.60 ± 0.50

The subscripts 1 and 2 indicate the solvent-monomer activation parameter, which as stated before is the dominant one in the semidilute zone, and any changes in the overall energy are influenced by γ_1,2_ values during the fitting procedure, while the other components are considered constant. This is maintained accordingly for the concentrated zone with γ_2,2_. In the fitting for the dilute zone, both components are maintained constant, since such cases involve activation energies mostly due to the frictional forces of the solvent molecules. The pure solvent viscosity parameter, η_0_, provides the necessary solvent molecular interactions for consideration. From the information of Table 6, it can be seen that the overall γ varies around 4.00 kJ/mol (±0.50 kJ/mol) across all ranges of mole fractions for all of the molecular weights. Activation energies for different systems as reported in the literature^5,29^ are of the same magnitude for the viscous motion of such polymer solutions. The activation energies for the systems are very similar, even though there are differences in the monomer sizes of the different polymers. The expected HPC monomer size is almost 7–8 times larger than that of PMMA, PEG, PDMS, or PAN monomers. It implies that the activation energy of polymer solutions is not very dependent on the monomer size. The other well-known parameter of internal interaction of polymer solutions, the Flory–Huggins interaction parameter, also has very close values around 0.48 for the same polymer systems as studied by us. They vary by 5–10% in their values depending on the types of solvents. It is similar to our observations, even at higher concentrations of solutions, that simple molecular interactions are not the exact source for the activation energies. Water-soluble polymers especially often develop hydrophobic interactions, which strikingly lead to phase separation or a demixing on heating. This rather complex cooperative interaction induces an additional ordering of the water molecules in the immediate vicinity of hydrophobic groups. With semiflexible chains like the cellulose derivatives, long chains may be soluble, but short ones of the same substitution pattern unexpectedly become insoluble and tend to crystallize.^68^

## Conclusions

We have shown that the previously established nanoscale viscosity scaling form can be applied for macroscale viscosity analysis. Previous works on PEG/PEO-water and PDMS-ethyl acetate solutions can also be applied to other polymer solutions of different solvents. Furthermore, we have established the means to apply this analysis on the basis of the polydispersity of the polymer samples.

In our overall studies, two clear crossovers between the concentration regimes were observed, as represented by the c* and c**. These crossover points change the scaling parameters as well as concentration-dependent coil dimensions. Scaling parameter changes were of the same order as for the previously reported polymer systems. Coil dimension model was carefully developed previously^30^ and applied here appropriately. This allows us to guarantee that the effects of concentration changes, interchain and intrachain interations, repulsions, and screenings are portrayed in the resulting size and structure of the polymer-solvent systems.

Our scaling eq 4 provides a method for characterizing the macroscale viscosity of polymer solutions. It is applicable for a broad range of concentrations, molecular weights, temperatures, and polydispersities. Previously developed notions of viscous flow as an activated energy process^4,5,29,30^ have been successfully reevaluated to obtain information regarding the various components influencing the flow of complex systems. The proposed approach has been developed based on common polymer characterization notions: hydrodynamic and gyration radii, correlation length, and Flory exponents. All parameters were carefully interpreted and calculated while taking into consideration every variation due to concentration and temperature changes. Furthermore, through eqs 15–18, we expand its use for all types of standard and nonstandard-grade polymers. Most studies are based on highly monodispersed samples. However, synthesis of such monodispersed polymers at bulk amounts is impossible, and corresponding theories have few benefits in large-scale applications. Using commercially available polymers, the overall impact of obtaining a general viscosity scaling model is highly significant.

We developed our conclusions after performing precise viscosity measurements through accurate techniques for a number of model good solvent systems: HPC in water, PMMA in toluene, PAN in DMSO, and previously, PEG/PEO in water, and PDMS in ethyl acetate. Based on a previously well-established scaling model, this investigation shows that it can be further enhanced to cover more extensive complex systems. Literature data^5,6,29,30,50,63,69^ support the validity of our proposed physical approach, in conjunction with curves obtained from our own experimental results. We have already shown through extensive studies^2,3,7^ that such a model is crucial to the diffusive motion studies of nanoscale objects in complex systems. The current work completes our approach to establishing a uniform length-scale characterization method for different types of polymer systems with different purposes of application.

## Supporting Information Available

Viscosity versus concentration plots for used polymers (Figure S1); dynamic viscosity data obtained through rheometry for all of the polymers used (Tables S1–S5); rheological measurement information (Section S1); molar mass distribution plot (Figure S2); molecular weight distributions of used polymers obtained through GPC measurements (Figure S3); polydispersity obtained through GPC measurements (Table S6); GPC measurement information (Section S2); hydrodynamic radius of the polymers obtained through DLS measurements (Figure S4); DLS measurement information (Section S3); individual fitting of HPC and PAN with *M*_w_ and *M*_v_ (Figures S5 and S6); and individual fitting information (Section S4) (PDF)
