# Supplementary material for: Macroscopic Viscosity of Polymer Solutions from the Nanoscale Analysis
Source: ACS Appl Polym Mater. 2021 Apr 12;3(5):2813–22. doi: 10.1021/acsapm.1c00348 (PMC8159165; doi:10.1021/acsapm.1c00348)
Supplement: Supplementary file 1 — ap1c00348_si_001.pdf [file ap1c00348_si_001.pdf]

Supporting Information for Macroscopic viscosity of polymer solutions from the nanoscale analysis

Airit Agasty,[†,‡] Agnieszka Wisniewska,[†] Tomasz Kalwarczyk,[†] Kaloian Koynov,[‡] and Robert Holyst*,[†]

†*Department of Soft Condensed Matter, Institute of Physical Chemistry, Polish Academy of Science, Kasprzaka 44/52, 01-224 Warsaw, Poland*

‡*Max Planck Institute for Polymer Research, Ackermannweg 10, 55128 Mainz, Germany*

E-mail: rholyst@ichf.edu.pl

Phone: +48 22 343 3123.

S1. Rheological Measurements

Viscosity measurements were performed using a Malvern Kinexus Pro rheometer and a Bohlin Gemini rheometer with a cone-plate and cylinder-cylinder geometries. Temperature range used for the measurements were 283-323 K. The tables below (Tables S1-S5) depict the variation of the viscosity with change in concentrations at different molecular weights and different temperatures. Fig S1 below represents the plots of the same.

S2. Molecular weight distribution - GPC

Gel Permeation Chromatography (GPC) measurements were performed to obtain the molecular weight distribution and thereby the polydispersity index of each polymer. Such a mass distribution is usually of the form shown in Fig S2. Measuring apparatus and its configu-

Table S1: Dynamic Viscosity η of HPC80k at different temperatures and concentrations.

Concentration	Temperature				
g/ml	283 K	288 K	293 K	298 K	303 K
-	η (Pa s)				
0.017	-	0.008	0.007	0.006	-
0.025	0.016	0.015	0.013	0.012	0.010
0.033	0.026	0.022	0.020	0.018	0.016
0.050	0.070	0.060	0.049	0.040	0.036
0.067	-	0.129	0.107	0.092	-
0.100	-	0.599	0.538	0.352	-
0.150	1.298	1.254	1.098	1.013	0.980
0.200	-	1.974	1.973	1.747	1.736
0.225	2.853	3.167	2.687	2.871	2.841

Table S2: Dynamic Viscosity η of HPC100k at different temperatures and concentrations.

Concentration	Temperature				
g/ml	283 K	288 K	293 K	298 K	303 K
-	η (Pa s)				
0.017	0.012	0.010	0.010	0.010	0.009
0.033	0.048	0.039	0.033	0.029	0.026
0.067	0.320	0.270	0.224	0.191	0.166
0.100	0.993	0.837	0.695	0.595	0.526
0.150	4.980	4.117	3.416	2.971	2.677
0.200	8.500	6.981	5.942	5.330	5.108
0.300	27.462	24.826	24.091	24.033	24.583

Table S3: Dynamic Viscosity η of PMMA24k at different temperatures and concentrations.

Concentration	Temperature				
g/ml	283 K	288 K	293 K	298 K	303 K
-	η (Pa s)				
0.100	0.002	0.002	0.002	0.002	0.002
0.200	0.011	0.011	0.010	0.010	0.010
0.300	0.064	0.061	0.061	0.059	0.059
0.400	0.235	0.228	0.214	0.218	0.218
0.500	0.688	0.548	0.595	0.565	0.514

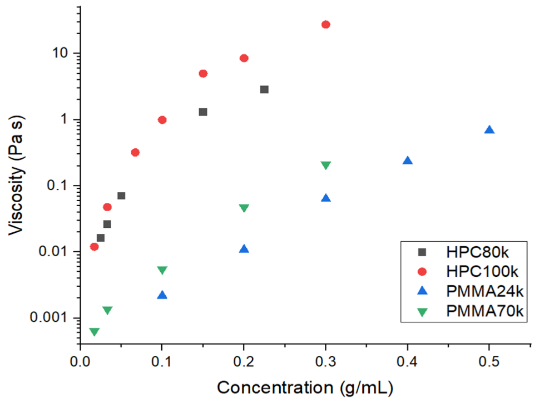

(a) 283K

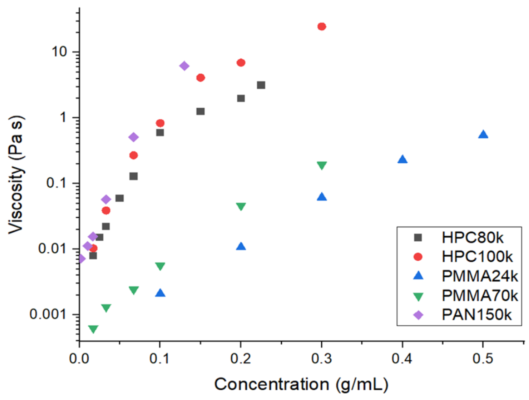

(b) 288K

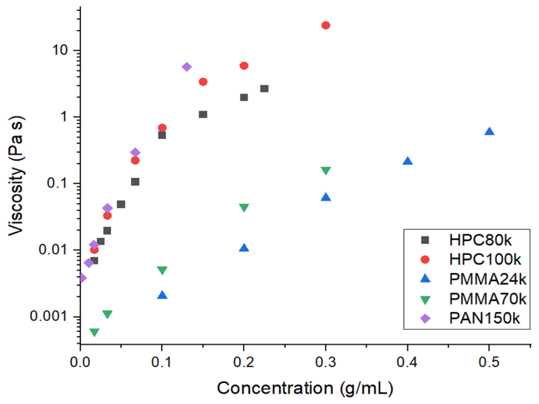

(c) 293K

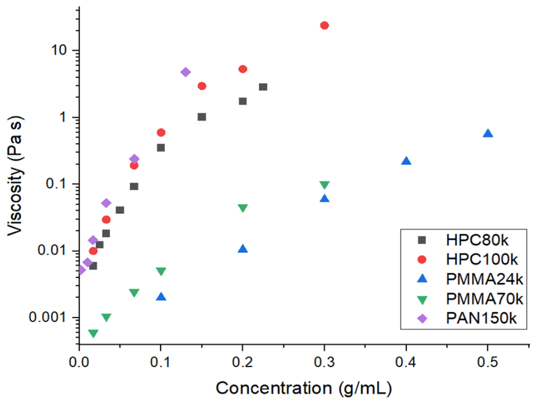

(d) 298K

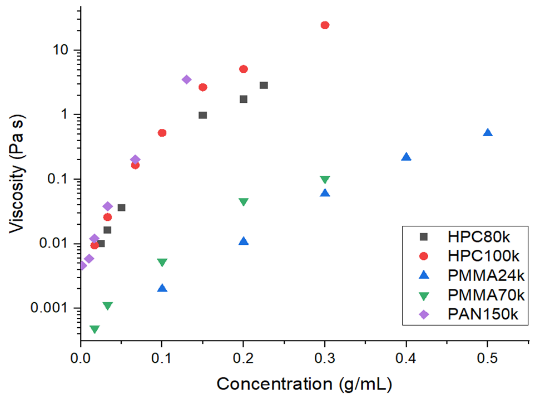

(e) 303K

Figure S1: Viscosity versus concentration plots for HPC, PMMA and PAN solutions at temperatures 283-303 K.

Table S4: Dynamic Viscosity η of PMMA70k at different temperatures and concentrations.

Concentration	Temperature				
g/ml	283 K	288 K	293 K	298 K	303 K
-	η (Pa s)				
0.017	0.0006	0.0006	0.0006	0.0006	0.0005
0.033	0.0013	0.0013	0.0011	0.0010	0.0010
0.067	-	0.0024	-	0.0024	-
0.100	0.0056	0.0056	0.0052	0.0051	0.0050
0.200	0.0476	0.0460	0.0453	0.0454	0.0460
0.300	0.2111	0.1941	0.1630	0.1005	0.1023

Table S5: Dynamic Viscosity η of PAN150k at different temperatures and concentrations.

Concentration	Temperature			
g/ml	288 K	293 K	298 K	303 K
-	η (Pa s)			
0.002	0.007	0.006	0.005	0.004
0.010	0.011	0.006	0.007	0.006
0.017	0.016	0.012	0.014	0.012
0.033	0.058	0.052	0.043	0.038
0.067	0.509	0.296	0.239	0.204
0.100	6.221	5.728	4.819	3.524

ration: The solvent used for such measurements was the same as for the rheological measurements. GPC measurements were performed with an Agilent Series 1260 device equipped with a PSS SECcurity pump and a PSS SECcurity RI refractive index detector. For the cellulose, 0.1M NaCl-water solution was used as an eluent at a flow rate of 1.0 mL/min and at a temperature of 303 K . For the PAN and PMMA, dimethyl formamide (DMF) and toluene, respectively, were used as an eluent at a flow rate of 1.0 mL/min and at a temperature of 333 K and 303 K respectively.

The obtained molecular weight distributions can be seen in Fig S3 below. The polydispersities and resultant molecular weights are provided in Table S6 below.

Table S6: Molecular weight distribution and polydispersities obtained from Gel Permeation Chromatography.

Polymer Grade	M_w (g/mol)	Polydispersity
HPC80k	107336	4.71
HPC100k	146690	4.82
PMMA24k	25063	1.08
PMMA70k	73416	1.14
PAN150k	333257	2.45

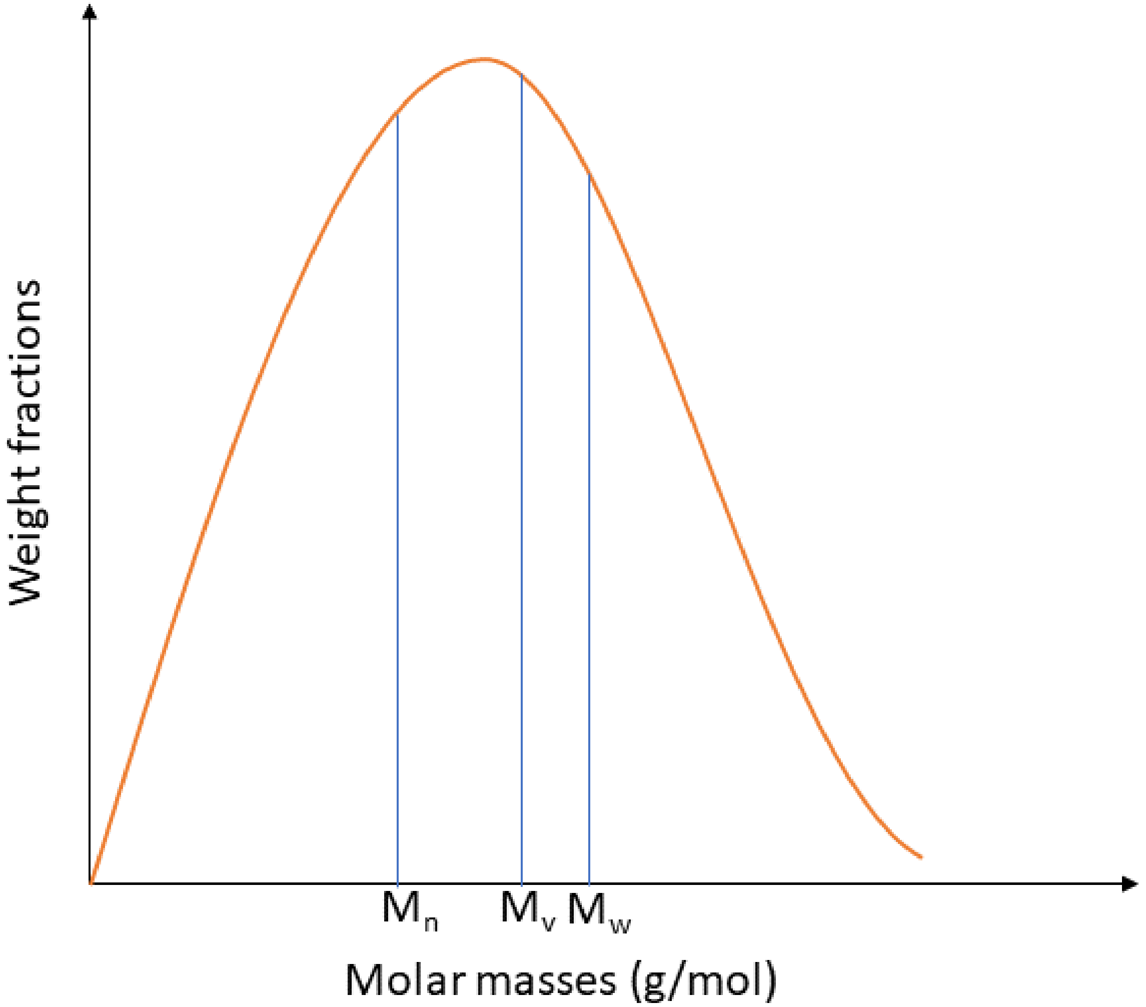

Figure S2: Molar mass distributions

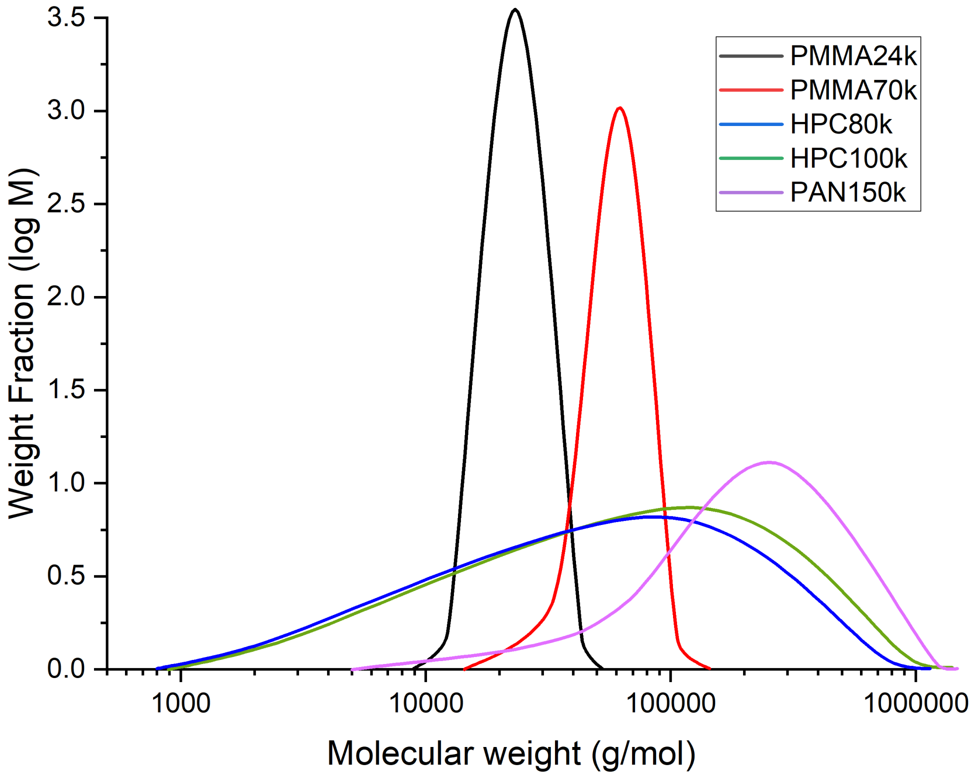

Figure S3: Molecular weight distributions of HPC, PMMA and PAN, measured at 298K by GPC.

S3. Dynamic Light Scattering data

Hydrodynamic radius, R_h, of the polymer coils were measured through Dynamic Light Scattering technique. Dilute polymer solutions of a specific concentration were used for each polymer molecular weight, and measurements were performed at 293-298 K for all of them. A Malvern Zetasizer Nano equipment was utilized for this purpose. The obtained mean volume size distribution for each polymer variety are shown in Fig S4 below.

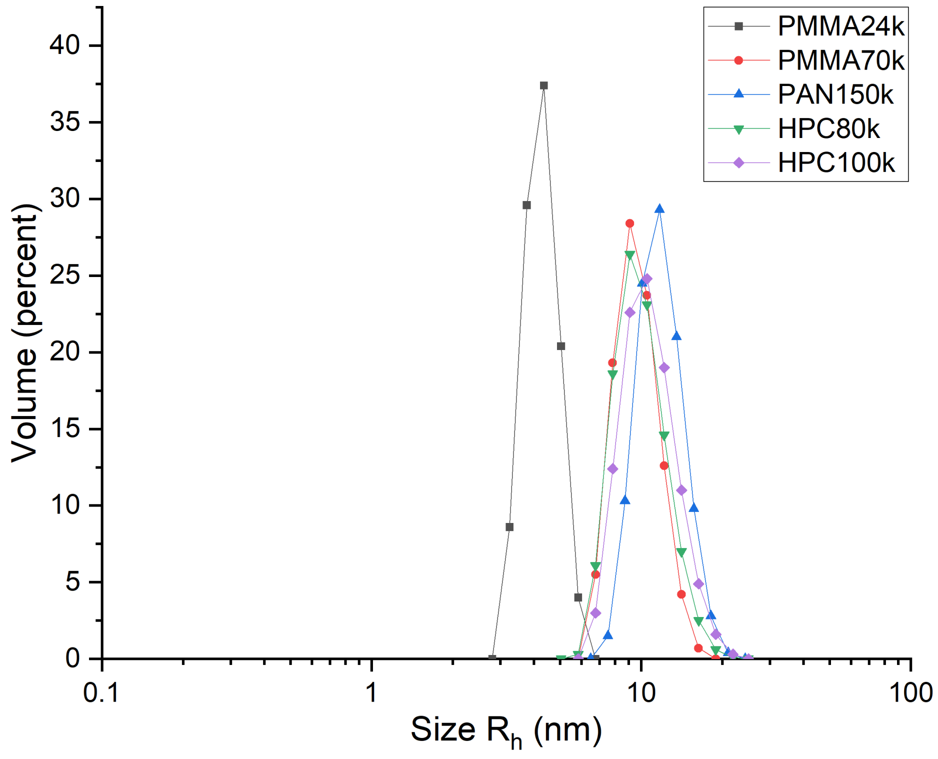

Figure S4: Hydrodynamic radius, R_h, measured at 293-298 K for all different molecular weights of HPC, PMMA and PAN through DLS.

S4. Individual fitting plots of HPC and PAN with M_w and M_v.

To further show the influence of M_w and M_v on the fitting, the results of HPC and PAN shown in Figs 2 and 4 of the main manuscript are provided here in greater detail, in the following Figs S5-S6.

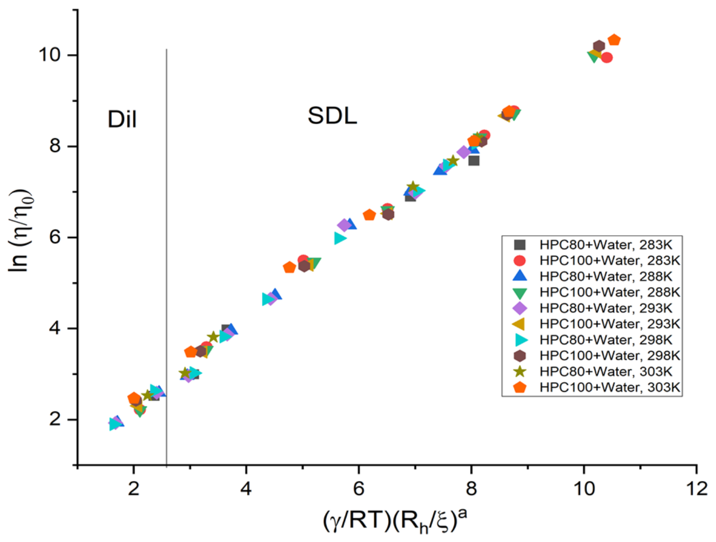

(a) HPC-water

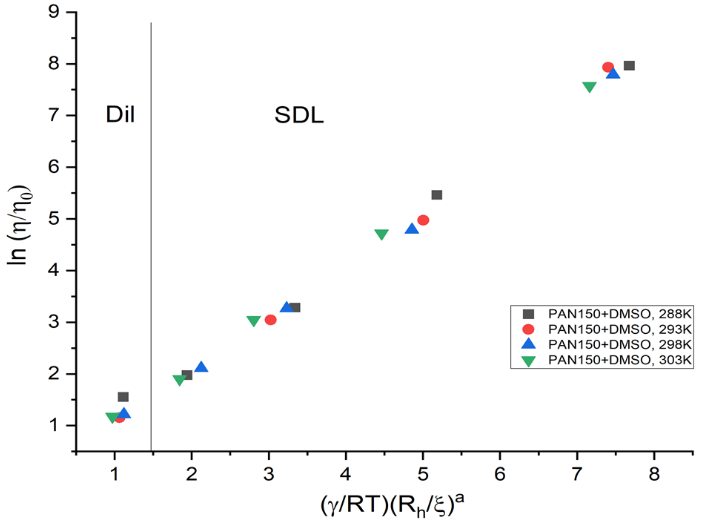

(b) PAN-DMSO

Figure S5: Viscosity scaling plots for all molecular weights of (a)HPC-water and (b)PAN-DMSO at temperatures of 283-303 K, plotted against M_w.

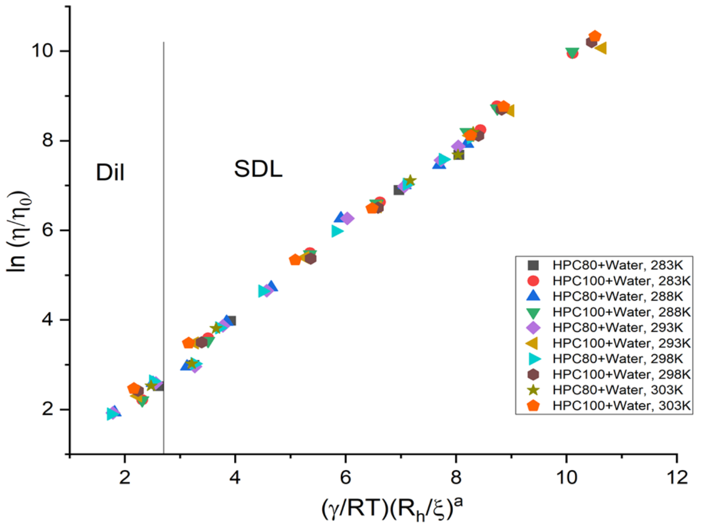

(a) HPC-water

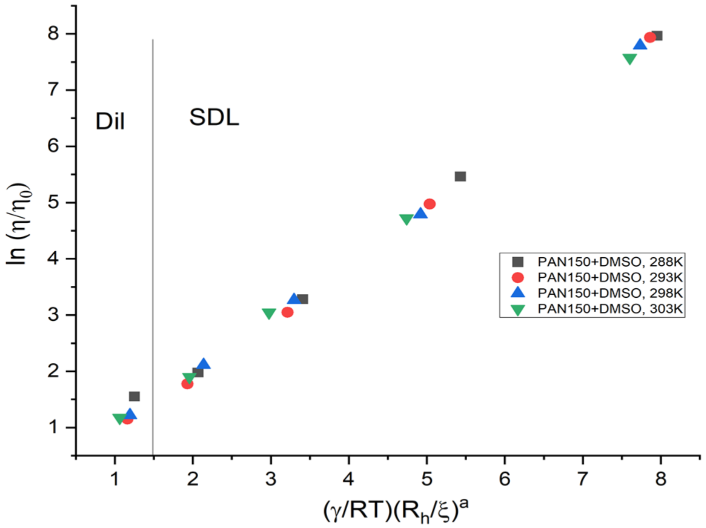

(b) PAN-DMSO

Figure S6: Viscosity scaling plots for all molecular weights of (a)HPC-water and (b)PAN-DMSO at temperatures of 283-303 K, plotted against M_v.
